# Overexpression of *eis* without a mutation in promoter region of amikacin- and kanamycin-resistant *Mycobacterium tuberculosis* clinical strain

**DOI:** 10.1186/s12941-018-0285-6

**Published:** 2018-07-16

**Authors:** Angkanang Sowajassatakul, Therdsak Prammananan, Angkana Chaiprasert, Saranya Phunpruch

**Affiliations:** 1 Department of Biology, Faculty of Science, King Mongkut’s Institute of Technology Ladkrabang, Bangkok, 10520 Thailand; 2 Tuberculosis Research Laboratory, National Center for Genetic Engineering and Biotechnology, National Science and Technology Development Agency, Pathum Thani, 12120 Thailand; 3 Research Affairs, Faculty of Medicine Siriraj Hospital, Mahidol University, Bangkok, 10700 Thailand; 4 Bioenergy Research Unit, Faculty of Science, King Mongkut’s Institute of Technology Ladkrabang, Bangkok, 10520 Thailand

**Keywords:** Tuberculosis, Drug resistance, Aminoglycoside, Efflux pump, *eis*, *whiB7*

## Abstract

**Background:**

Aminoglycosides such as amikacin and kanamycin are effective injectable second-line drugs for treatment of multidrug-resistant tuberculosis. Molecular mechanisms underlying aminoglycoside resistance are not well understood. We have previously identified the amikacin- and kanamycin-resistant *M. tuberculosis* MT433 clinical strain, of which all known mutations related to resistance have not been found. Drug efflux pump is one of reported resistance mechanisms that might play a role in aminoglycoside resistance.

**Methods:**

The expression levels of sixteen putative efflux pump genes, including *eis* and one regulator gene, *whiB7*, of MT433 in the presence of kanamycin were determined using the reverse transcription-quantitative PCR method. The effects of upregulated genes on amikacin and kanamycin resistance were investigated by overexpression in *M. tuberculosis* H37Ra strain.

**Results:**

Upon kanamycin exposure, other than *whiB7* and *eis* that were found extremely overexpressed, two drug efflux pump genes, namely Rv1877 and Rv2846c, showed specifically high-level of expression in *M. tuberculosis* MT433 strain. However, direct effect of overexpressed Rv1877 and Rv2846c on amikacin and kanamycin resistance could not be demonstrated in *M. tuberculosis* H37Ra overexpressed strain.

**Conclusions:**

Our finding demonstrated that overexpression of *eis* could occur without any mutations in the promoter region and be detectable in clinical isolate. This might be a consequence of overexpressed *whiB7*, resulting in amikacin and kanamycin resistance in *M. tuberculosis* MT433 strain.

**Electronic supplementary material:**

The online version of this article (10.1186/s12941-018-0285-6) contains supplementary material, which is available to authorized users.

## Background

Tuberculosis (TB) is a critical problem for public health in human worldwide. There are approximately 10.4 million global incidence cases of TB in 2016 (140 cases per 100,000 population) and most TB cases have been found in South-East Asian region (about 45%) [1]. Thailand is estimated as one of the top 20 high TB and TB/HIV burden countries between year 2016–2020. There are approximately 172 TB incidence cases per 100,000 population in Thailand in 2016 [1]. The emergence of drug resistance tuberculosis remains a significant cause of morbidity and mortality that leads to global problems including treatment and control of TB in many countries. Multidrug-resistant TB (MDR-TB) is defined as TB resistant to at least two most effective first-line antituberculosis drugs (isoniazid and rifampicin) and extensively drug-resistant TB (XDR-TB) is defined as MDR-TB additionally resistant to any fluoroquinolones and one of injectable second-line drugs (amikacin, kanamycin or capreomycin) [2]. In Thailand, approximately 6.8 MDR-TB incidence cases per 100,000 population were found in 2016 [1].

Amikacin (AMK) and kanamycin (KM) are aminoglycoside drugs generally used to treat MDR-TB [3]. The drugs bind to bacterial 16S rRNA in 30S ribosomal small subunit, resulting in an inhibition of protein synthesis [3, 4]. Three known resistance mechanisms of these drugs have been reported in *M. tuberculosis*. The major resistance mechanism is the modification of drug binding site at adenine base in position 1401 of *rrs* gene [5, 6]. The second mechanism is up-regulation of *eis* (enhanced intracellular survival), aminoglycosides modifying enzymes (acetyltransferases) encoding gene, that has been shown to confer a low-level kanamycin resistance [7]. The last mechanism is an overexpression of efflux pump genes that leads to a low-level resistance of antibiotics such as isoniazid, fluoroquinolones and aminoglycosides, in *Mycobacterium smegmatis* and *Mycobacterium tuberculosis* [8–12].

Efflux pump is a transmembrane protein, which is capable of export antibiotics and toxic compounds out of the cells [13]. There are at least five known families of drug efflux pump in bacteria including major facilitator superfamily (MFS), ATP binding cassette (ABC) transporter family, multidrug and toxic compound extrusion (MATE) family, small multidrug resistance (SMR) family and resistance-nodulation-division (RND) superfamily [14, 15]. In *M. tuberculosis*, the energy metabolism and ATP production from proton motive force (PMF) determine the drug susceptibility [16]. Tap (encoding by Rv1258c), the putative efflux pump, has been shown to play a role in aminoglycoside resistance in *M. tuberculosis* [17, 18].

Our previous study revealed that AMK- and KM-resistant *M. tuberculosis* clinical strains, MT433 and MT164, showed high-level resistance to AMK and KM (MIC > 64 µg/ml). None of point mutations in all known resistant genes, including *rrs*, *eis* promoter region, *tap*, and *whiB7*, has been reported in *M. tuberculosis* MT433 strain whereas A1401G mutation of *rrs* gene has been found in *M. tuberculosis* MT164 strain [19]. It is possible that expression level of genes, particularly genes encoding drug efflux pump, might play a significant role in the resistant phenotype of this MT433 strain. The present study, therefore, aimed to investigate the expression levels of 16 genes encoding putative efflux pumps and hypothetical predicted transmembrane proteins, *eis* and also *whiB7*, in *M. tuberculosis* MT433 and MT164 using the reverse transcription-quantitative PCR (RT-qPCR) method. Effects of overexpressed genes on AMK and KM susceptibility were validated in *M. tuberculosis* H37Ra strain.

## Methods

### Mycobacterial strains and culture conditions

Amikacin- and kanamycin-resistant *M. tuberculosis* MT433 and *M. tuberculosis* MT164 strains were obtained from the Drug-Resistant Tuberculosis Research Fund, Siriraj Foundation, Faculty of Medicine Siriraj Hospital, Mahidol University, Bangkok, Thailand. The *M. tuberculosis* MT433 was isolated from sputum of a male patient, 26 years of age who got a treatment at Maharaj Nakhon Ratchasima hospital, Nakhon Ratchasima province, Thailand. Draft genome sequence of *M. tuberculosis* MT433 was analyzed and submitted to the NCBI under Accession No. LGAX00000000 [20]. *M. tuberculosis* MT164 was previously characterized and found to carry the mutation in the *rrs* A1401G gene [19]. Mycobacteria were cultured on Löwenstein-Jensen (LJ) medium (BBL, USA) [21] and cultures were incubated at 37 °C for 3–4 weeks.

### Isolation of total RNA

One loopful of *M. tuberculosis* MT433 and MT164 cells were suspended in 500 μl of 10 mM Tris–HCl buffer containing 1 mM EDTA (pH 8.0). Cells were harvested by centrifugation at 12,000×*g* for 2 min. Cell pellet was resuspended in 1 ml of Middlebrook 7H9 medium [22] containing 6 µg/ml of KM. The cell mixture was incubated at 37 °C for 1 h before harvesting cells by centrifugation. Total RNA was isolated using TRIzol reagent (Invitrogen, USA) [23]. One millilitre of TRIzol reagent was added into the cell pellet. The mixture was mixed by vortexing for 15 s and then incubated on ice for 15 s. This procedure was done three times. Subsequently, total RNA was purified using PureLink^®^ RNA Mini Kit (Ambion, USA). The residual DNA in RNA was removed by adding DNaseI (Invitrogen, USA) with a final concentration of 0.05 U/µl. The mixture was incubated at 37 °C for 1 h. The concentration of total RNA was determined by measuring an absorbance at 260 nm using NanoDrop™ 2000/2000c Spectrophotometer (Thermo Fisher Scientific, USA).

### Complementary DNA (cDNA) synthesis

The cDNA synthesis was performed using the RevertAid First Strand cDNA Synthesis Kit (Thermo Fisher Scientific, USA). RNA was reverse-transcribed in 20 µl reaction mixture containing Transcriptor reverse transcriptase buffer (50 mM Tris–HCl (pH 8.3), 50 mM KCl, 4 mM MgCl_2_ and 10 mM DTT), 10 U of protector RNase Inhibitor (Promega, USA), 0.25 mM each dNTP (Invitrogen, USA), 2 µM RT primer (Additional file 1: Table S1), 1 µg of RNA and 10 U of Transcriptor reverse transcriptase (Thermo Fisher Scientific, USA). The RT-PCR reaction mixture was incubated at 42 °C for 30 min and reaction was stopped by incubating at 85 °C for 5 min.

### Determination of genes expression using reverse transcription quantitative real-time PCR (RT-qPCR)

The RT-qPCR reaction was performed by PrimeScript™ RT Master Mix (Takara, USA) using PikoReal™ 24-well Real-time PCR System (Thermo Fisher Scientific, USA) according to the manufacturer’s protocol. The *sigA* gene encoding the major sigma factor of RNA polymerase that is constitutively expressed in *M. tuberculosis* [24] was used as a reference gene for normalization of gene expression in this study. Briefly, 20 µl of reaction mixture comprised PrimeScript RT Master Mix (10 mM Tris–HCl (pH 8.3), 15 mM KCl, 1.6 mM MgCl_2_) containing 2 mM each dNTP 10 µM each primer (Additional file 1: Table S1), 0.2 µg of cDNA and 4 U of PrimeScript Reverse Transcriptase (Takara, Japan). The qPCR condition consisted of an initial denaturation at 95 °C for 5 min, followed by 45 cycles of amplification with denaturation at 95 °C for 20 s, annealing at 60 °C for 30 s and extension at 72 °C for 20 s. The 2^−ΔΔCq^ calculation method was used for the relative quantification of gene expression as previously described (Cq is the quantification cycle or the cycle number at which a threshold amount of amplicon DNA is produced) [25]. The ΔCq is the cycle difference between Cq of target gene and Cq of reference gene (*sigA*) (control) (ΔCq = Cq _target gene _− Cq _reference gene_). The ΔΔCq is the cycle difference between ΔCq of sample (KM exposed cells) and ΔCq of calibrator (KM unexposed cells) (ΔΔCq = ΔCq _sample _− ΔCq _calibrator_). The 2^−ΔΔCq^ value is a relative quantification of gene expression. When compared relative gene expressions of target genes in cells with and without KM exposure conditions, expression level above 1 was considered as “increased expression” and expression level equal to or above 4 was considered as “overexpression” [25–28].

### Construction of Rv1877 and Rv2846c overexpressed in *M. tuberculosis* H37Ra strains

Rv1877 and Rv2846c genes were amplified by PCR using specific primers (Forward Rv1877-BamHI 5´-GGATCCATGGCGGGCCCCA-3´ and Reverse Rv1877-BamHI 5´-GGATCCCTACGTTGTAGCCGCGA-3´ for Rv1877 amplification and Forward Rv2846c-BamHI 5´-GGATCCATGACGGCTCTCAACGACAC-3´ and Reverse Rv2846c-BamHI 5´-GGATCCTTACAGCTCGCCGGCGTCGA-3´ for Rv2846c amplification). PCR was conducted in 50 µl reaction mixture containing 20 mM Tris–HCl (pH 8.4), 50 mM KCl, 1.5 mM MgCl_2_, 200 µM each dNTP, 0.5 µM each primer, 50 ng of genomic DNA and 2.5 U of *Taq* DNA polymerase (Promega, USA). The PCR condition consisted of an initial denaturation at 94 °C for 5 min, followed by 35 cycles of denaturation at 94 °C for 30 s, annealing at 60 °C for 1–2 min and extension at 72 °C for 7 min. PCR products were purified using QIAquick PCR Purification Kit (Qiagen, Germany). The amplified products were submitted to DNA sequencing for confirming the accuracy of nucleotide sequences and directions. The amplified genes were then subcloned into the plasmid pSMT1, which is a replicative plasmid carrying a hygromycin resistance cassette [29], by replacing the *luxAB* gene in sense and antisense directions, resulting in the recombinant plasmids pSMT1-Rv1877(S), pSMT1-Rv1877(AS), pSMT1-Rv2846c(S), and pSMT1-Rv2846c(AS). The recombinant plasmids were transformed into *M. tuberculosis* H37Ra by electroporation. Briefly, *M. tuberculosis* H37Ra competent cells were prepared by culturing bacteria until OD_600_ reached approximately 0.75. Cells were harvested by centrifugation at 5000×*g* for 10 min at 4 °C and washed three times with ice-chilled 10% glycerol before centrifugation again. The cell pellet was finally resuspended in 1/100 original culture volume of ice-chilled 10% glycerol. One µg of recombinant plasmid DNA was added into 100 µl of competent cells. DNA was introduced into the competent cells by electroporation using Gene Pulser (Bio-Rad, USA) with a pulse controller setting at voltage 2.5 kV, capacitor 25 µF and resistance 1000 Ω. Positive transformants were selected on Middlebrook 7H10 agar containing 10% Oleic acid-albumin-dextrose-catalase (OADC) and 50 µg/ml of hygromycin.

### Drug susceptibility testing and determination of minimal inhibitory concentration (MICs)

The susceptibility testing was performed by agar dilution method on Middlebrook 7H10 agar (Difco, USA) supplemented with 10% OADC [30]. The MICs value of AMK and KM were determined on agar containing 50 µg/ml of hygromycin and a single concentration of each drug (0, 4, 8, and 16 µg/ml) (Sigma Aldrich, Germany). The MICs were defined as the lowest concentration of AMK and KM that prevented visible growth of mycobacteria after incubation for 3–4 weeks at 37 °C.

## Results

### Expression levels of efflux pump genes upon kanamycin exposure in *M. tuberculosis* MT433 and MT164

**Fig. 1:**
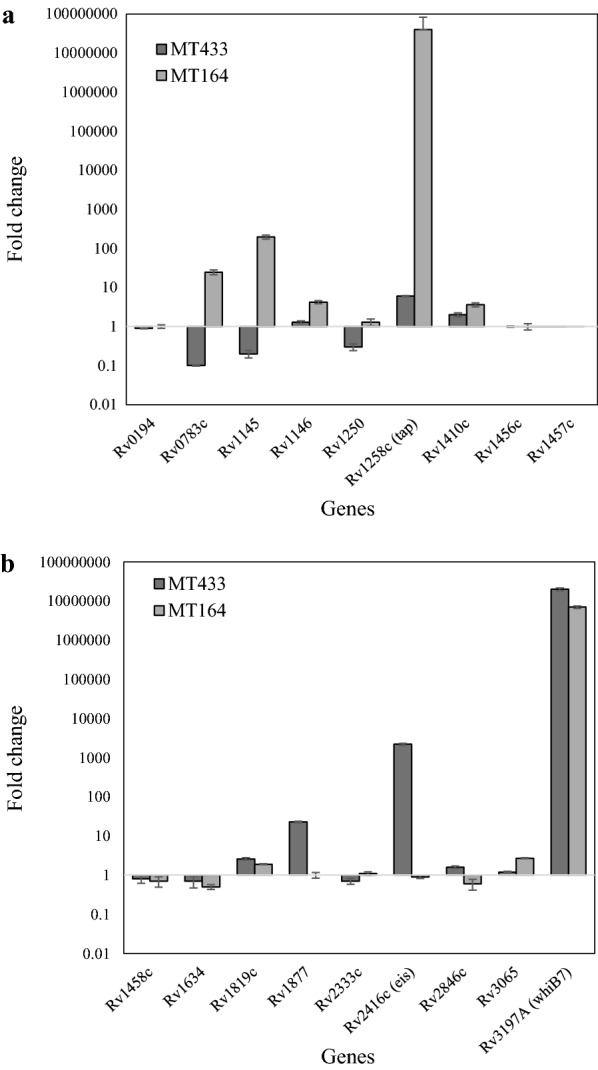
The relative fold change of the expression levels of genes, Rv0194, Rv0783c, Rv1145, Rv1146, Rv1250, Rv1258c (tap), Rv1410c, Rv1456c, Rv1457c (**a**), Rv1458c, Rv1634, Rv1819c, Rv1877, Rv2333c, Rv2416 (*eis*), Rv2846c, Rv3065 and Rv3197A (*whiB7*) (**b**) in kanamycin-resistant *M. tuberculosis* MT433

The expression levels of 16 efflux pump and hypothetical transmembrane protein encoding genes, aminoglycoside acetyltransferase (*eis*) gene, and transcription regulator *whiB7* were determined in *M. tuberculosis* MT433 (KM-resistant strain with unknown mechanism) and *M. tuberculosis* MT164 (KM-resistant strain with *rrs* A1401G mutation) after exposure to 6 µg/ml of KM by RT-qPCR. The expressions of all target genes upon KM exposure were compared with those of reference gene and those of unexposed controls. Results revealed that KM could induce expression of several genes with different manners (Table 1 and Fig. 1). Three genes (Rv1877, Rv2846c, and Rv2416c) were specifically upregulated in *M. tuberculosis* MT433 upon KM exposure but they were downregulated or slightly increased in *M. tuberculosis* MT164 strain. In addition, four genes (Rv0783c, Rv1250, Rv2333c, and Rv1145) were specifically upregulated in *M. tuberculosis* MT164 upon KM exposure but these genes were downregulated in another strain. Three genes (Rv1634, Rv1457c and Rv1458c) showed a downregulation in both strains. Interestingly, *eis* (Rv2416c) was found significantly overexpressed only in *M. tuberculosis* MT433 whereas *tap* (Rv1258c) was much higher overexpressed in MT164 strain than MT433 strain. The transcriptional regulator *whiB7* (Rv3197A) involving in aminoglycoside resistance was extremely up-regulated in both strains.

**Table 1:** Differential expression of genes investigated upon kanamycin exposure in *M. tuberculosis* MT433 and MT164 strains

Genes	Differential expression fold change (2^−ΔΔCq^)	
	MT433	MT164
Rv0194	0.9	1.0
Rv0783c	0.1	24.7
Rv1145	0.2	195.0
Rv1146	1.3	4.2
Rv1250	0.3	1.3
Rv1258c (*tap*)	6.0	4.0 × 10^7^
Rv1410c	2.0	3.6
Rv1456c	1.0	1.0
Rv1457c	0.0	0.0
Rv1458c	0.8	0.7
Rv1634	0.7	0.5
Rv1819c	2.6	1.9
Rv1877	22.8	1.0
Rv2333c	0.7	1.1
Rv2416c (*eis*)	2.2 × 10^3^	0.9
Rv2846c	1.6	0.6
Rv3065	1.2	2.7
Rv3197A (*whiB7*)	2.0 × 10^7^	7.0 × 10^6^

### Construction of overexpressed *M. tuberculosis* H37Ra strains

In order to determine whether the significantly high upregulated efflux pump encoding genes, Rv1877 and Rv2846c, could directly affect the AMK and KM susceptibility, recombinant plasmids pSMT1 carrying each Rv1877 and Rv2846c with different directions, sense (S) and antisense (AS), were constructed and transformed into *M. tuberculosis* H37Ra. Four or eight transformants of each overexpressed *M. tuberculosis* H37Ra strains, namely H37Ra::Rv1877(S), H37Ra::Rv1877(AS), H37Ra::Rv2846c(S), and H37Ra::Rv2846c(AS) were selected for further investigation. The susceptibility of AMK and KM was investigated in all overexpressed strains, including *M. tuberculosis* H37Ra::pSMT1 control strain.

### Susceptibility of overexpressed *M. tuberculosis* H37Ra strains to amikacin and kanamycin

The minimal inhibitory concentrations (MIC) of AMK and KM were determined in all overexpressed strains. Results revealed that neither overexpressed *M. tuberculosis* H37Ra strains carrying Rv1877 nor Rv2846c could confer either AMK or KM resistance as summarized in Table 2.

**Table 2:** MICs of amikacin and kanamycin of overexpressed *M. tuberculosis* H37Ra strains

Strains	MIC (µg/ml)	
	Amikacin	Kanamycin
H37Ra	4	4
H37Ra::pSMT1	4	4
H37Ra::Rv1877(S)	4	4
H37Ra::Rv1877(AS)	4	4
H37Ra::Rv2846c(S)	4	4
H37Ra::Rv2846c(AS)	4	4

## Discussion

In this study, the expression levels of 16 efflux pump genes, *eis*, and *whiB7* were investigated upon kanamycin exposure in *M. tuberculosis* MT433 (resistant strain with unknown mechanism) and the resistant control *M. tuberculosis* MT164 (strain with *rrs* A1401G mutation), and the effect of overexpressed genes on AMK and KM susceptibility was determined by performing the gene overexpression experiments in *M. tuberculosis* H37Ra strain. Previous study reported that overexpression of *eis* resulting from mutations located at the promoter region is the second most common cause of KM resistance [7]. Eis is a mycobacterial effector that is related to the survival within human macrophage [31, 32] and suppression of *eis* made the anaerobic persistent bacilli susceptible to the aminoglycoside antibiotics [33]. In addition, the enhanced *eis* expression due to the specific mutations in promoter region which exhibited a low-level KM resistance in KM-resistant *M. tuberculosis* clinical isolates [7, 34, 35]. Similarly, high expression level of *eis* (Rv2416c) has been detected in our study (Table 1 and Fig. 1), suggesting that *eis* might be related to phenotypic KM resistance in *M. tuberculosis* MT433 even if this strain did not contain any mutations in the promoter region [19]. Concordantly, the *whiB7* (Rv3197A) was also found significantly overexpressed in this strain and even in the resistant strain with *rrs* mutation (Table 1 and Fig. 1). WhiB7, an inducible putative transcriptional regulator, has been shown to regulate several genes including *eis* and *tap*; both are involved in aminoglycoside resistance [36, 37]. Our results indicated that overexpression of *whiB7* resulted in significant overexpression of *eis* only in the MT433 strain but not in MT146 strain, suggesting that overexpression of *eis* is not necessary in case of strain carrying *rrs* mutation. However, the mechanism underlying high overexpression of *whiB7* and *eis* without any mutations at promoter region occurred in this MT433 clinical strain remains inconclusive.

Another possibility that might be responsible for drug resistance is the drug efflux mechanism. High-level expression of two specific genes in the major facilitator superfamily (MFS), Rv1877 and Rv2846c (*efpA*), was found only in the MT433 strain. It is possible that overexpression of these two genes is also involved in AMK and KM resistance. Previous studies described several secondary transporters capable of exporting cation compounds and antibiotics including antituberculosis drugs, such as isoniazid, rifampicin and aminoglycosides [38, 39]. The efflux pump protein Rv0783c, Rv1250, Rv1258c (Tap), Rv1410c (P55), Rv1634, Rv1877, Rv2333c, Rv2846c and Rv3239c were also categorized into the MFS family [33]. Deletion of Rv1877 homologue in *M. smegmatis* (58% identity to *M. tuberculosis* Rv1877) increased susceptibility to erythromycin, novobiocin, tetracycline, and KM [40]. Our findings did not show any direct evidences suggesting that Rv1877 is related to AMK and KM resistance because overexpression of this gene in *M. tuberculosis* H37Ra could not increase the MICs against AMK and KM (Table 2). Previous study showed that deletion of *efpA* (Rv2846c) homologue has been shown to increase susceptibility to ethidium bromide and acriflavine but it decreased susceptibility to rifampicin, isoniazid and chloramphenicol in *M. smegmatis* [24]. No evidence has been reported for this gene in *M. tuberculosis*. However, overexpression of Rv2846c (*efpA*) did not confer AMK and KM resistance (Table 2). Results indicated that both Rv1877 and Rv2846c did not directly affect the susceptibility of the MT433 strain to AMK and KM. The remaining genes, excepting for Rv1258c and Rv1410c that were found overexpressed in both MT433 and MT164 strains, were found down-regulated in the MT433 strain.

On the other hand, four specific genes, Rv0783c (*emrB*), Rv1145, Rv1250, and Rv2333c (*stp*), were significantly upregulated in the MT164 strain that contains A1401G mutation in *rrs* whereas seven genes, namely Rv1146, Rv1258c (*tap*), Rv1410c, Rv1456c, Rv1819c (*bacA*), Rv3065 (*mmr*) and Rv3197A (*whiB7*), were found overexpressed in both strains (Table 1 and Fig. 1). Rv1145 and Rv1146 are classified into the resistance-nodulation-division (RND) family that showed 62% homologous to *mmpL* of *M. smegmatis* [40]. Although their nucleotide sequences showed homologous to the *M. smegmatis mmpL* efflux pump gene, they did not function as a drug efflux pump in *M. tuberculosis* [40]. Rv1456c, Rv1457c and Rv1458c, the ATP-binding cassette (ABC) superfamily genes, have been found overexpressed in the presence of ethambutol, isoniazid, rifampicin, and streptomycin in *M. tuberculosis* [41]. Rv2333c has been reported to associate with aminoglycoside resistance in *M. tuberculosis* clinical isolates [42]. However, it is unclear whether in a strain harboring a well-characterized mutation conferring resistance, like MT164, the increasing expression level of efflux pump genes still plays a role in the resistant phenotype.

## Conclusions

In this study, the high expression level of *eis* was found in *M. tuberculosis* MT433 whereas the downregulation of *eis* was found in *M. tuberculosis* MT164. An unusual resistance mechanism was firstly described, by which overexpression of *eis* without any mutations in the promoter region was identified by the reverse transcription quantitative real-time PCR. It should be considered that any resistant *M. tuberculosis* strains caused by this mechanism could not be detected by all currently available diagnostic tests for detection of drug resistance. Our finding provides a new insight that could benefit for developing a new test with higher sensitivity. In addition, several drug efflux genes were found selectively overexpressed in the presence of drug either in strains with or without a known mutation conferring resistance. However, the exact role of these efflux genes could not be demonstrated in the present study.

## Additional file

**Additional file 1: Table S1.** Primers used in this study.
