# Supplementary material for: Overexpression of eis without a mutation in promoter region of amikacin- and kanamycin-resistant Mycobacterium tuberculosis clinical strain
Source: Ann Clin Microbiol Antimicrob. 2018 Jul 16;17:33. doi: 10.1186/s12941-018-0285-6 (PMC6047124; doi:10.1186/s12941-018-0285-6)
Supplement: Supplementary file 1 — Additional file 1: Table S1. Primers used in this study. [file 12941_2018_285_MOESM1_ESM.docx]

**Table S1** Primers used in this study

| **Genes** | **Primers^a^** | **Sequences (5´-3´)** | **Tm (˚C)** | **Product sizes (bp)^b^** |
| --- | --- | --- | --- | --- |
| Rv0194 | RT-Rv0194  F Rv0194  R Rv0194 | 5´-TGTGGGCGCCGTCCTCGACA-3´  5´-CGATGCCACCGATGCCCAGG-3´  5´-GGCCTCGGTGGCAGGATCCA-3´ | 60  59  58 | 225 |
| Rv0783c | RT-Rv0783c  F Rv0783c  R Rv0783c | 5´-GAATGCCGCGGGGATCAGCG-3´  5´-TGGGCATGGGCATGGGCTGC-3´  5´-CGAGGAAGGGTCAACCGCCG-3´ | 60  60  59 | 254 |
| Rv1145 | RT-Rv1145  F Rv1145  R Rv1145 | 5´-TCGGGAACAGCGCAGTCGCC-3´  5´-GGCGGCTCGGCGATGGAGTA-3´  5´-CGATAGCCAACGCGAGGCCC-3´ | 61  60  60 | 262 |
| Rv1146 | RT-Rv1146  F Rv1146  R Rv1146 | 5´-CCATTGCGCGTCAGCGAGGC-3´  5´-GCCGCCAACGACGAGAGCGT-3´  5´-GCTCATGCAGCCACGCCAGG-3´ | 62  61  61 | 256 |
| Rv1250 | RT-Rv1250  F Rv1250  R Rv1250 | 5´-GCGGCTGGGTGTCGCAATCC-3´  5´-ACCTGGCCGGTGCGAGCTCA-3´  5´-AGGGCGACGAAGGCGGGCAA-3´ | 59  61  61 | 241 |
| Rv1258c (*tap*) | RT-Rv1258c  F Rv1258c  R Rv1258c | 5´-GAGCCGATCCTACGGGCCGA-3´  5´-GGTCATCGCGTTCCTGCCGC-3´  5´-GGCATGCAGTCCAGCGGCGT-3´ | 59  60  61 | 226 |
| Rv1410c | RT-Rv1410c  F Rv1410c  R Rv1410c | 5´-ACAGACCTAGCACCGCGCCG-3´  5´-TGCCGGCGATGCACACCGAC-3´  5´-AGGCTGGCGTTGGGCGGGAT-3´ | 61  60  61 | 244 |
| Rv1456c | RT-Rv1456c  F Rv1456c  R Rv1456c | 5´-GGCACCGGCCACGTGAATGG-3´  5´-ACGGGCACGCTAGTCACGGC-3´  5´-AACCTGGCCGCGGTGCTGGT-3´ | 58  60  61 | 209 |
| Rv1457c | RT-Rv1457c  F Rv1457c  R Rv1457c | 5´-GCCCCACACCGCTAGGACGA-3´  5´-TCGGCTTTGCGCTCGGCTGG-3´  5´-CGCCGTCGGGATCACGTTCG-3´ | 58  62  60 | 212 |
| Rv1458c | RT-Rv1458c  F Rv1458c  R Rv1458c | 5´-CTGCCGCAACTTCCTGCCGG-3´  5´-GCGATGGCGTGACCGTGGTG-3´  5´-ACCTGCGGGTCAACCGGACC-3´ | 60  59  60 | 268 |
| Rv1634 | RT-Rv1634  F Rv1634  R Rv1634 | 5´-CAACGCGGTAAGCGCCGGTC-3´  5´-GGTGATGGCGTCGGGGTTGG-3´  5´-TGACCACCACACCGGCCAGC-3´ | 60  58  58 | 251 |
| Rv1819c | RT-Rv1819c  F Rv1819c  R Rv1819c | 5´-TTACACTTCGGCGGGCGCCG-3´  5´-CACGCTGACCAAGGTGGCGC-3´  5´-TGCAGTCCGGCAGCTCGCTG-3´ | 61  60  62 | 221 |

**Table S1** Primers used in this study (continued)

| **Genes** | **Primers^a^** | **Sequences (5´-3´)** | **Tm (˚C)** | **Product sizes (bp)^b^** |
| --- | --- | --- | --- | --- |
| Rv1877 | RT-Rv1877  F Rv1877  R Rv1877 | 5´-GAACGAGCACCGCGTCGGTG-3´  5´-TGCCGAACGGGGTGCGACTG-3´  5´-GGGAGTCGACCTGACGGTGC-3´ | 60  60  57 | 261 |
| Rv2333c | RT-Rv2333c  F Rv2333c  R Rv2333c | 5´-GCGCACTCGACGGTCATCGC-3´  5´-CCGCCTGGCTTTCGGCCACA-3´  5´-CAGTGGCGACGAGCAACGCC-3´ | 61  61  61 | 219 |
| Rv2416c (*eis*) | RT-Rv2416c  F Rv2416c  R Rv2416c | 5´-CACTGGCAAACGCCGCGTCG-3´  5´-GGACCCGTTACCCCACCTGC-3´  5´-CTGCCGCATCGGTCGGGGTA-3´ | 61  56  60 | 224 |
| Rv2846c | RT-Rv2846c  F Rv2846c  R Rv2846c | 5´-ACAGCTCGCCGGCGTCGATC-3´  5´-TGGCCGTCGTCCCGCTGACT-3´  5´-CCCACAGCAGGCCGTAGGTG-3´ | 62  60  59 | 240 |
| Rv3065 | RT-Rv3065  F Rv3065  R Rv3065 | 5´-GGCACCCGCCAGGTTCAACG-3´  5´-CTCTTGTGCGCGATCTTCGCGG-3´  5´-GGCGACCAGCACAATGGCGG-3´ | 58  63  60 | 210 |
| Rv3197A (*whiB7*) | RT-whiB7  F whiB7  R whiB7 | 5´-GCATCCTTGCGCGGACGTCC-3´  5´-CTGACAGTCCCCAGACAGACCC-3´  5´-CACCACCCCAAACGCCCCAG-3´ | 60  57  57 | 196 |

^a^ RT primers used for cDNA synthesis; F and R primers used for real-time PCR

^b^ Products size of real-time PCR
